# Development of a Novel Multifunctional Cementitious-Based Geocomposite by the Contribution of CNT and GNP

**DOI:** 10.3390/nano11040961

**Published:** 2021-04-09

**Authors:** Mohammadmahdi Abedi, Raul Fangueiro, António Gomes Correia

**Affiliations:** 1 Department of Civil Engineering, ISISE, University of Minho, 4800-058 Guimarães, Portugal; mohammadmehdi.abedi@gmail.com (M.A.); agc@civil.uminho.pt (A.G.C.); 2 Department of Mechanical Engineering, University of Minho, Campus de Azurém, 4800-058 Guimarães, Portugal

**Keywords:** self-sensing, stabilized sand, CNT/GNP, mechanical, microstructural, durability, piezoresistivity

## Abstract

In this study, a self-sensing cementitious stabilized sand (CSS) was developed by the incorporation of hybrid carbon nanotubes (CNTs) and graphene nanoplatelets (GNPs) based on the piezoresistivity principle. For this purpose, different concentrations of CNTs and GNPs (1:1) were dispersed into the CSS, and specimens were fabricated using the standard compaction method with optimum moisture. The mechanical and microstructural, durability, and piezoresistivity performances, of CSS were investigated by various tests after 28 days of hydration. The results showed that the incorporation of 0.1%, 0.17%, and 0.24% CNT/GNP into the stabilized sand with 10% cement caused an increase in UCS of about 65%, 31%, and 14%, respectively, compared to plain CSS. An excessive increase in the CNM concentration beyond 0.24% to 0.34% reduced the UCS by around 13%. The addition of 0.1% CNMs as the optimum concentration increased the maximum dry density of the CSS as well as leading to optimum moisture reduction. Reinforcing CSS with the optimum concentration of CNT/GNP improved the hydration rate and durability of the specimens against severe climatic cycles, including freeze–thaw and wetting–drying. The addition of 0.1%, 0.17%, 0.24%, and 0.34% CNMs into the CSS resulted in gauge factors of about 123, 139, 151, and 173, respectively. However, the Raman and X-ray analysis showed the negative impacts of harsh climatic cycles on the electrical properties of the CNT/GNP and sensitivity of nano intruded CSS.

## 1. Introduction

Among all monitoring methods, self-sensing composites with intrinsic stress, strain, and damage sensing capabilities based on piezoresistivity provide a more integrated, real-time, and practical solution for infrastructure damage detection, considering geomaterial properties and nature. Although several studies have evaluated self-sensing in various composites, such as concrete, cement paste, polymer, and asphalt [1,2,3,4,5], little research exists that assesses the impact of this method in geomaterials. Self-sensing composites arise from the dispersion of the conductive phase in the non-conductive composite [6,7,8]. These conductive components form a conducting electrical network within the composites. When the composites are subjected to strain, stress, or any other external factors, this conducting network is disturbed, leading to a change in the electrical resistivity [9,10,11,12]. However, the sensitivity and performance of self-sensing composite are affected by some factors such as electrode status, current, temperature, humidity, and loading, among which the type of conductive phase, their concentration, and distribution have particular importance [13,14,15,16]. Although various types of fibrous and nanomaterials have been used as the conductive phase in the cementitious matrix, carbon nanomaterials (CNMs) have attracted more attention due to their unique electrical, physical, and mechanical properties [17,18,19,20,21]. Furthermore, reinforcing cementitious composites by highly dispersed CNMs with low concentration can improve their mechanical, microstructural, and durability performance [10,22,23,24,25,26]. Recently, studies have been conducted on the synergic effects of carbon nanotubes (CNTs) and graphene nanoplatelets (GNPs) on the mechanical, microstructural, durability, and piezoresistivity performances of cementitious mortar using compatible, affordable, and effective dispersion techniques [5,27,28]. These studies reported that hybrid CNTs/GNPs can improve percolation and electron tunneling effects, which increase the conductive path and, consequently, reduce the percolation threshold significantly. Hence, the synergic effects of CNTs/GNPs also decrease the required concentration of conductive filler and eliminate concerns about rising production costs and porosity formation. Accordingly, it is possible that hybrid CNTs/GNPs combinations can also achieve similar performance in cementitious stabilized sand (CSS). Taking this into account, this study aims to utilize the advantages of the synergic effects of these CNMs in order to advance existing knowledge by developing a novel piezoresistive self-sensing cementitious geocomposite. In this route, different concentrations of hybrid CNT/GNP (1:1) were incorporated into the mixture of the sand and 10% cement (by weight of the sand). The optimum moisture of different nano-intruded mixture was measured and cylindrical specimens were fabricated using the standard compaction method. The mechanical performance of specimens was evaluated by unconfined compressive strength (UCS) tests. Microstructural properties of CNM-reinforced CSS were also investigated by various tests. The durability of the specimens was investigated against hybrid climatic cycles, including a combination of both freeze–thaw and drying–wetting cycles, which represents harsh climatic conditions. In addition, the piezoresistivity behaviors of nano-intruded specimens were evaluated using the four probes method under cyclic compression loading. To investigate the sustainability and long-term performance of this novel self-sensing CSS, the effects of water content and severe climatic cycles on its piezoresistivity properties were also evaluated.

The outcomes of this study provide an extensive contribution to the new era of smart cementitious geocomposites, with primary applications in transport and energy infrastructure, such as roller-compacted concrete dams, rammed earth, ground improvement, and, in particular, pavement structural layers and critical zones, including transition zones.

## 2. Materials and Methods

### 2.1. Raw Materials

**Figure 1:**
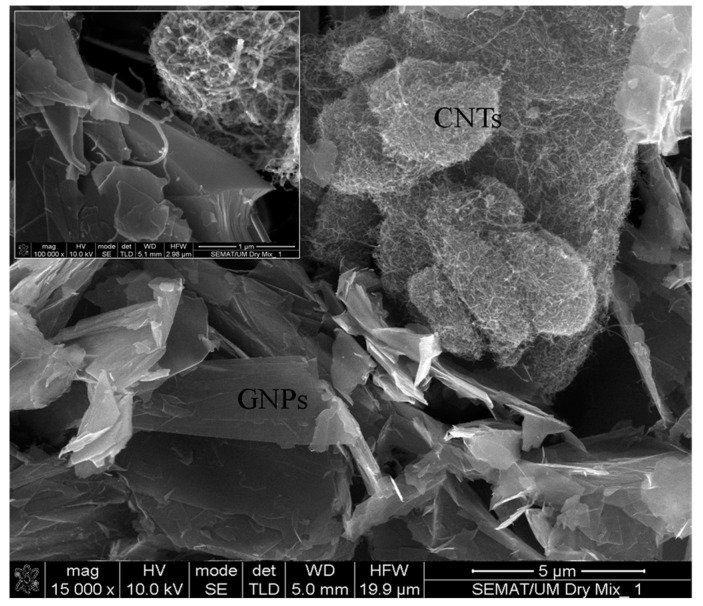
CNT and GNP morphology (dry mix).

A multi-layer type of GNP and multi-wall CNT (MWCNT) with purities of approximately 99.5% and 98%, respectively, were utilized as conductive fillers in this study. The characteristics of the GNPs and MWCNTs are summarized in Table 1 [27,28]. The morphology of hybrid GNPs/CNTs in the dry mix state was also investigated by scanning electron microscopy (SEM), as depicted in Figure 1.

For hybrid CNT/GNP dispersion, Pluronic F-127 (PF-127) was used as a noncovalent surfactant. This surfactant is a nonionic triblock copolymer surfactant and has a specific bipolar molecule structure [28]. In addition, tributyl phosphate 97% (TBP) with ½ of the surfactant weight ratio was used as an antifoaming agent to prevent porosity formation caused by surfactant function, following previous research [28,29].

The sand used for this study was CEN Standard sand, with siliceous and clean particles that were classified as well-graded sand according to the Unified Soil Classification System (USCS). The physical properties of the sand are presented in Table 2. The ordinary Portland cement type I (CEM I 42.5R) was also used as a binder to prepare the CSS.

### 2.2. CNT and GNP Dispersion Method

Nowadays, a compatible and effective method for hybrid CNT/GNP dispersion in aqueous suspension, to be used in multifunctional cementitious composites, has been developed. In this technique, high dosages of CNT/GNP (1%; by the water volume, (1:1)) were dispersed by 10% PF-127 (by weight of CNMs) with the addition of 50% TBP (by weight of surfactant) via 3 h of sonication (80 W output power and 45 kHz frequency) at 40 °C [28]. A similar method was used for CNT/GNP dispersion [28].

**Figure 2:**
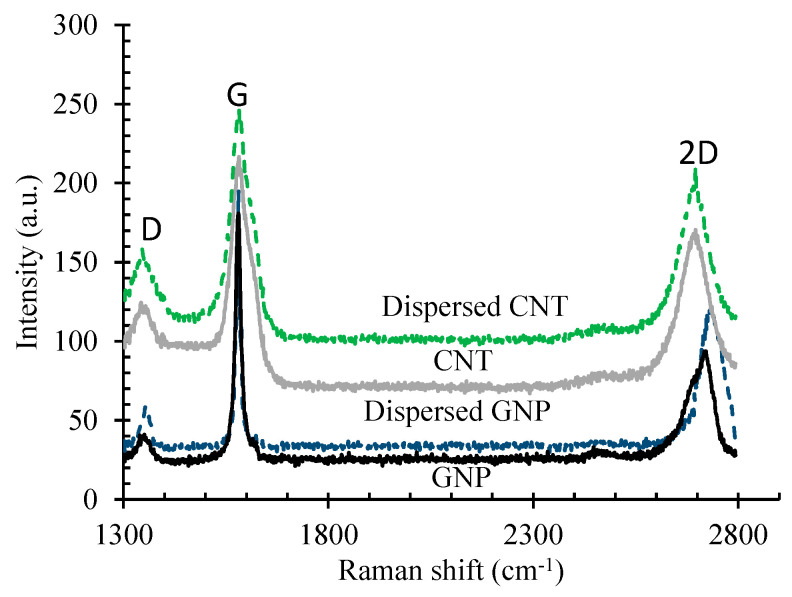
Raman analysis results of CNTs and GNPs.

Under these specific mixing conditions, negligible structural damage was expected for the carbon nanomaterials. Raman spectroscopy (Figure 2) was also carried out on CNMs using laser excitation with a wavelength of 532 nm to ensure the absence of adverse effects on the structural qualities of CNTs and GNPs, such as edge-type defects, a reduction in aspect ratio, and sp^2^ domain crystallinity (La), which have a deleterious influence on their mechanical and electrical properties [30,31].

### 2.3. CSS Fabrication

**Figure 3:**
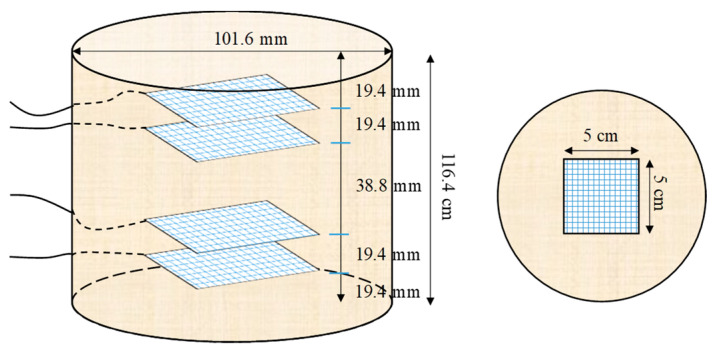
Representation of the specimen’s geometrical and electrode layout.

In stabilized sands, the cement content usually varies by around 10% owing to the target strength of the sand–cement [32,33]. In this study, 10% cement concentration (by weight of the sand) was also utilized in order to fabricate CSS. First, the cement and sand were added to a steel bowl and blended with a stainless steel blade at a rotational speed of 140 rpm for 1.5 min. Then, CNM suspensions composed of 0.1%, 0.17%, 0.24%, and 0.34% CNT/GNP (by weight of the sand and equal portions, 1:1), prepared by ω_opt_ (for each CNM concentration), were sprayed into the mixture and blended at a 285 rpm higher speed for another 2.5 min. Thereafter, the mixture was poured into a plastic bag to prevent moisture loss. The CSS cylindrical specimens were fabricated by the compaction method according to the ASTM D698 compaction standard. Split molds with dimensions of 101.6 × 116.4 mm were filled with the wet mixture in three equal-height layers. A calculated amount of the well-mixed wet mixture was measured (to an accuracy of 0.01 g), poured into the split mold, and then compacted carefully by a metal tamper to the desired height (controlled by a caliper to an accuracy of 0.02 mm). After the molds were filled with the compacted mixture, they were sealed at both ends and extracted after a 24 h period for hardening; then, the specimens were cured underwater for 28 days. For the specimens that were used for piezoresistivity tests, four square meshes of copper with dimensions of 50 mm × 50 mm were embedded as electrodes at distances of 38.8 and 19.4 mm from the middle of the specimens (Figure 3).

The amount of the mixture moisture after compaction was also measured to ensure the lack of water evaporation. Sample identification was conducted based on the variation of CNM concentrations in such a way that the specimens GC (0.1%), GC (0.17%), GC (0.24%), and GC (0.34%) were composed of 0.1%, 0.17%, 0.24%, and 0.34% CNT/GNP (by weight of dry sand), respectively. In order to evaluate the water content effects on piezoresistivity behavior of the nano-intruded specimens, two specimens were made with different water content and the same CNM concentration as specimen GC (0.34%). These specimens, labelled GC (0.34 + 2%w) and GC (0.34−2%w), contained 2% more and 2% less water than the optimum content, respectively.

### 2.4. Mechanical and Microstructural Characterization

The unconfined compressive strength test was used following the ASTM/D2166M standard for the evaluation of mechanical properties of cylindrical specimens. The results were calculated by the mean of at least 3 specimens. Because the modulus of elasticity for cementitious geomaterials is typically expressed by the modulus at 50% of the peak stress [34,35,36,37], the Tangent E _(50%)_ of reinforced CSS by different CNM concentrations was calculated at 50% of the maximum compression stress.

**Figure 4:**
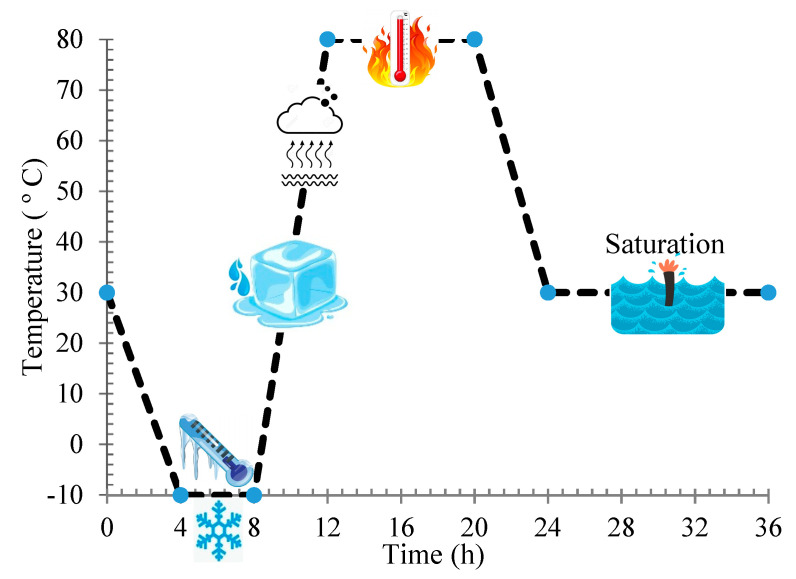
A schema of the climatic cycle protocol.

The effects of different CNM concentrations on maximum dry density and ω _opt_ were investigated according to the ASTM D697-78 standard. Thermal analysis and X-ray diffraction analysis (XRD) was conducted for the evaluation of the cement hydration process [27]. The specimens’ fracture surfaces were also investigated using scanning electron microscopy (SEM) and energy-dispersive X-ray spectrometry (EDX) [27,28]. Furthermore, an ultrasonic nondestructive test was performed for the microstructural investigation following the BS EN 12504-4 standard through two probes along the longitudinal axis. To evaluate the effects of the adversarial environmental conditions on the physical and electrical performances of the nano-intruded specimens, a hybrid climatic cycle, including both freeze–thaw and drying–wetting cycles, as illustrated in Figure 4, was used. This cycle represents the harsh climatic conditions in deserts. The percentages of the specimens’ weight loss and the ultrasonic wave passing time after 12 cycles were utilized as a criterion for determining the resistance of specimens to this climatic cycle. The quantitative approach, via Raman spectroscopy, was utilized to investigate the defect status of CNMs evolved under the effect of climatic cycles. Raman analysis was performed on dry CNTs and GNPs using an Ar ion laser with an excitation wavelength of a 514.5 nm (2.41 eV) at room temperature.

### 2.5. Piezoresistivity Measurement

To investigate the piezoresistivity performance of CNM-reinforced CSSs, the specimens with embedded electrodes were first dried at 70 °C for 72 h after 28 days of hydration to ensure the absence of moisture effects on electrical conductivity values. As can be seen in Figure 3, a four-probe technique was used in this study to measure the fractional changes in electrical resistivity (FCR) under cyclic axial compression loading. In this circuit, a reference resistor of 100 Ω was connected to the outer probes in series powered by a direct current (DC) source with a constant value of 20 V. Power was supplied for 40 min, and it took between 25 and 30 min to stabilize the supply in CNM-reinforced CSS. Two digital multimeters were used to measure the voltage variation of the two inner probes and resistor. Three cycles of 4 kN axial compression loading at a rate of 40 N/s were used in order to evaluate the CSS piezoresistivity behavior. The electrical resistivity *ρ*(*t*) of each sample was obtained from the average of three voltage measurements and calculated by combining first and second Ohm’s law equations (Equation (1) and Equation (2), respectively), as presented in Equation (3) [10]:

(1) $$R\left(t\right)=\frac{V_{1}\left(t\right)}{I\left(t\right)}=\frac{V_{1}\left(t\right)}{V_{2}\left(t\right)/100}$$

(2) $$R\left(t\right)=\rho \left(t\right)\frac{L}{A}$$

(3) $$\rho \left(t\right)=\frac{V_{1}\left(t\right)}{V_{2}\left(t\right)/100}\times \frac{A}{L}$$

where *V*_1_(*t*) and *V*_2_(*t*) are the inner probes and resistor voltage, respectively; *R*(*t*) is the resistance between the two inner probes; *I*(*t*) is the current between the outer electrodes; A is the contact surface between the electrode and CSS; and *L* is the spacing between the inner electrodes. For the following assessment of CSS piezoresistivity, the *FCR* was calculated by Equation (4):

(4) $$FCR=\frac{\rho \left(t\right)-\;\rho_{0}}{\rho_{0}}$$

where *ρ*_0_ is the initial electrical resistivity measured before loading and *ρ(t)* is the resistivity at time t during the test. To evaluate the sensitivity of CNT/GNP reinforced CSS, the gauge factor (*GF*) is also defined as the relative change in electrical resistivity over the strain (Equation (5):

(5) $$GF=\frac{FCR}{\varepsilon }$$

where *ε* is the applied strain along the force axis.

## 3. Results

### 3.1. Compaction Tests Results

**Figure 5:**
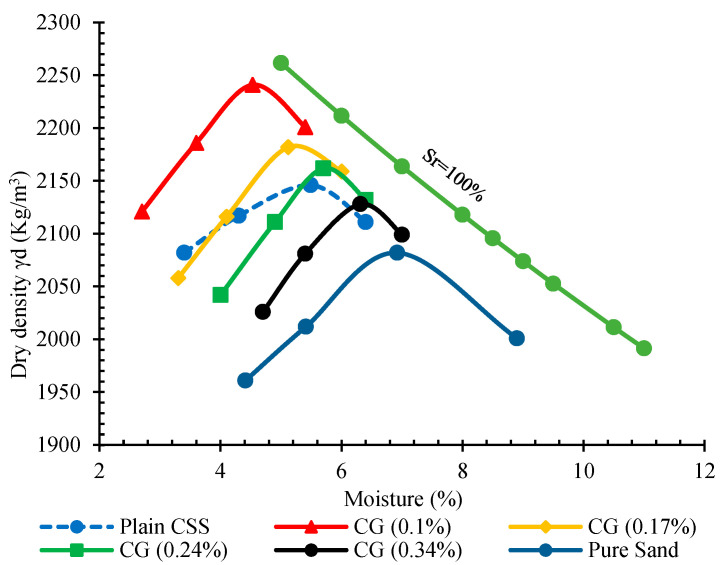
Compaction curves of plain and reinforced cementitious stabilized sand (CSS) by different carbon nanomaterial (CNM) concentrations.

The compaction curves of plain and reinforced CSS by different CNM concentrations are presented in Figure 5. As can be observed, the addition of 10% cement increased the maximum density of pure sand and reduced the optimum moisture.

**Figure 6:**
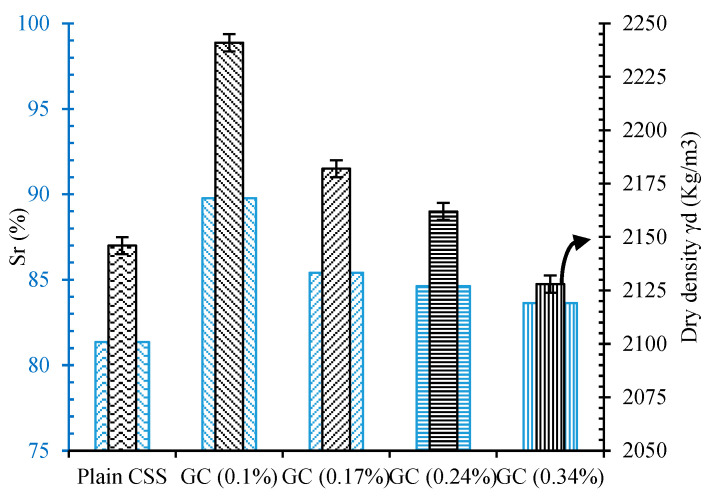
Maximum dry density and degree of saturation for plain and reinforced CSS with different CNM concentrations.

Reinforcing CSS by different concentrations of CNM also caused similar trends. The incorporation of 0.1% hybrid CNT/GNP increased the maximum dry density to around 2241 kg/m^3^. However, specimens composed of 0.17% and 0.24% CNM also showed higher maximum dry density; they were accompanied by a declining trend, such that an excessive increase in the CNM concentration beyond 0.24% led to a decrease in the maximum dry density compared to plain CSS. The results of the saturation degree (Sr) are also indicated in Figure 6.

A similar trend to that of the compaction results was also observed for the saturation degrees. The augmentation of the density affected by CNM reinforcement decreased the void ratio, which consequently led to an increase in saturation degrees. The maximum saturation degree of around 89% was achieved via the incorporation of 0.1% CNM into the plain CSS, and an excessive increase in CNM concentration beyond 0.24% caused a decrease in the saturation degree.

**Figure 7:**
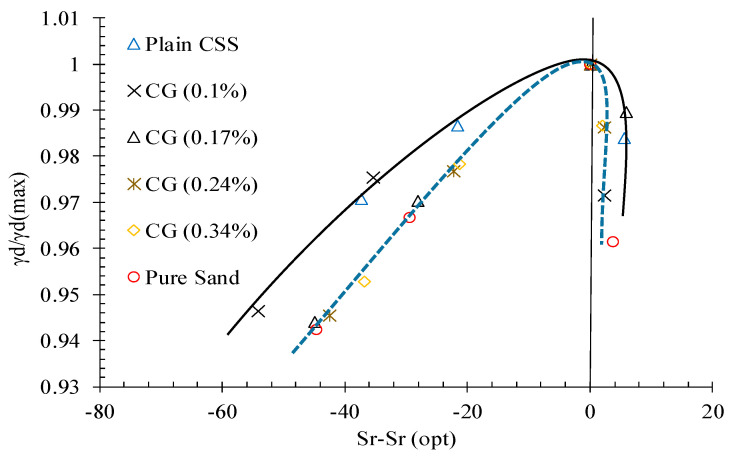
“γd/γd_(max)_” vs. “Sr-Sr _(opt)_” relationship.

An interesting relationship between “γd/γd_(max)_” and “Sr-(Sr)_opt_” appeared, as illustrated in Figure 7. A similar trend has been reported in recent studies [38], indicating these laboratory results can be applied directly in the field. This is a major issue that supports the use of these novel materials in practice.

**Figure 8:**
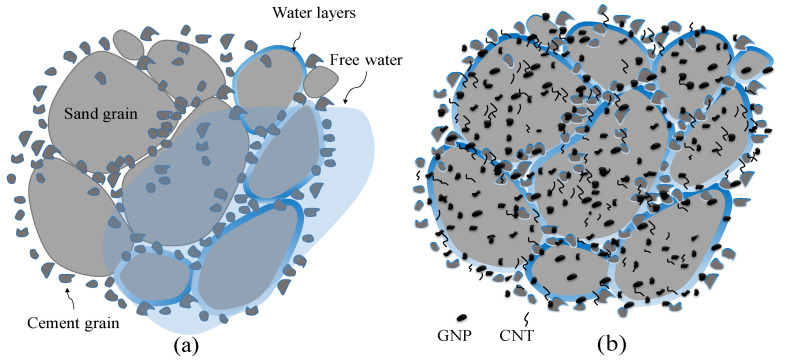
A schema of compacted: (**a**) Plain CSS, reinforced; (**b**) CSS by CNMs.

Although increases in maximum dry density caused by the addition of CNMs were less significant, optimum moisture reductions were considerable. Reinforcing CSS by 0.1% and 0.17% CNMs reduced the optimum moisture by about 21% and 14%, respectively, whereas excessive increases in the CNM percentage increased the optimum moisture by around 8% and 17% compared to plain CSS. However, the filler function of hybrid tubular CNTs and plate-shaped GNPs was not ineffective; this mechanism does not play a crucial role in the augmentation of the maximum dry density due to the low concentration of these nanomaterials. In fact, their role as a moisture regulator is more effective in increasing the slipperiness of cement and sand grains, and consequently, in CSS density improvement. CNTs and GNPs absorb and decrease the free water among the cement and sand grains, and also prevent the inhomogeneous distribution of water [29,39]. In addition, these nano-scaled particles with a water-impregnated surface and high aspect ratio encourage the slipping and latching of cement and sand grains together (Figure 8).

**Figure 9:**
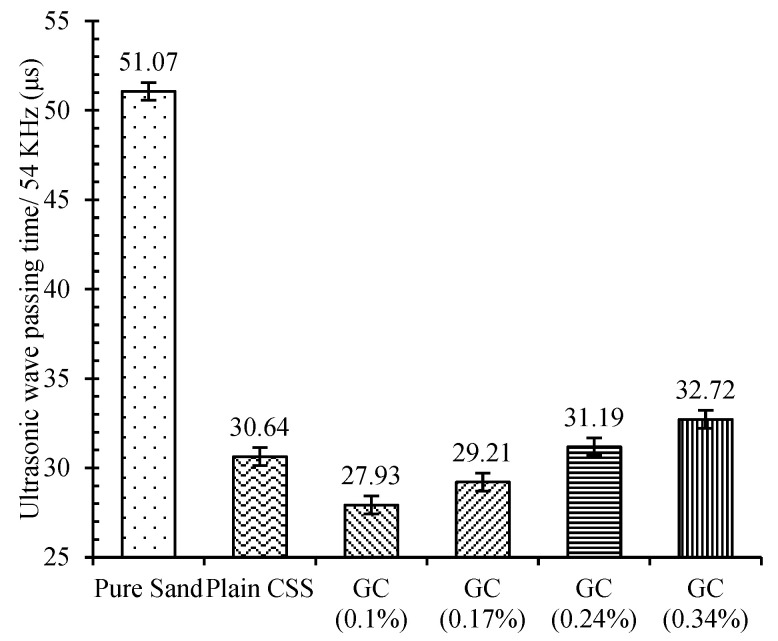
An ultrasonic wave passing time for plain and reinforced CSS with different CNM concentrations.

It should be noted that, in the case of high CNM concentrations, the required moisture increases abruptly, which can cause the gaps between the particles to reduce the maximum dry density. This amount of moisture is possibly converted to free water at high compaction energies. By starting the cement hydration process, the mechanical performance of CSS gradually increases due to the microstructural improvement. The results of ultrasonic non-destructive tests after a 28-day hydration period, as presented in Figure 9, also indicate this issue.

As can be observed, the ultrasonic wave passing time of compacted pure sand was reduced by about 40% by the addition of 10% cement after 28 days of curing. In the case of CNM-reinforced CSS, the passing times of the ultrasonic wave were also reduced by incorporating certain amounts of CNMs into the plain CSS due to a more cohesive microstructure, which was in line with the above compaction tests outcomes and the UCS results discussed in Section 3.3. The lower amount of ultrasonic wave passing time was achieved for CSS reinforced by 0.1% CNM, and an excessive increase in CNM concentration beyond 0.24% increased the passing time compared to that of plain CSS.

In general, the reduction in optimum water content and the increasing maximum dry density of the cementitious stabilized soil, as a result of different additions of CNMs, has frequently been reported. Alsharef et al. [40], reported a 13.6% decrease in optimum water content and a 1.5% increase in dry density by the dispersion of 0.075% MWCNTs into the clayey sand. For the soil sample reinforced by carbon nanofibers (CNFs), the maximum decrease in optimum water content was 14% at 0.075% CNFs content. Furthermore, dry density increased from 1.86 to 1.88 (g/cm^3^).

### 3.2. Microstructural Investigations

In general, reinforcing the cementitious composite by specific amounts of CNMs enhanced physical performance due to three main mechanisms, such as filler function, the bridging and/or divination of nano cracks, and the increasing of the cement hydration rate.

**Figure 10:**
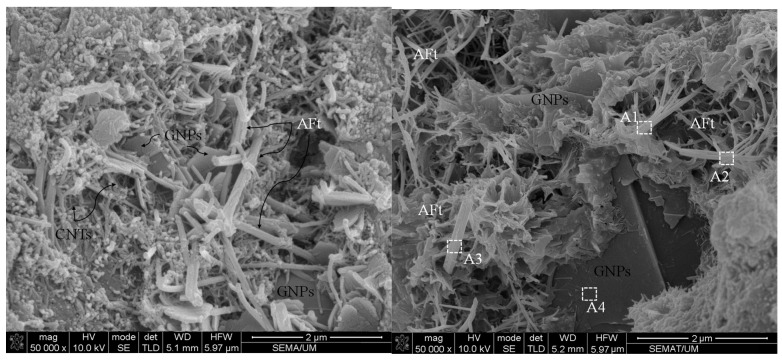
Scanning electron microscopy (SEM) images of the reinforced CSS by high CNM concentration CG (0.34%) (the areas with white markings are the areas selected for EDX analysis).

Although the incorporation of an optimum concentration of hybrid CNTs and GNPs with different 1D and 2D geometrical shapes makes it possible to fill wider ranges of porosities, among the hydration products that led to their reinforcement, a high concentration of CNMs caused the formation of agglomerations and, consequently, increased the amount of porosities (Figure 10).

**Figure 11:**
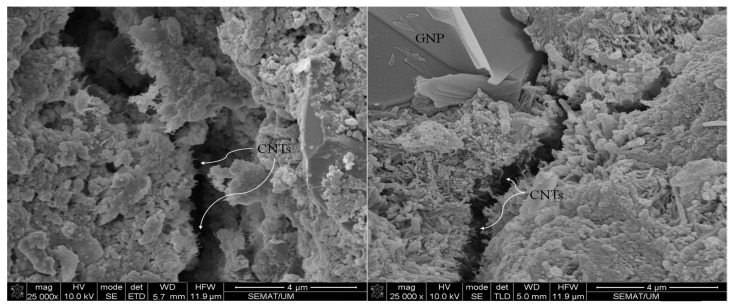
SEM morphology of CNTs and GNPs crack bridging and deviation mechanism in CNM-reinforced CSS.

In addition, the EDX results, which are listed in Table 3, indicate that the needle-shaped crystals around the agglomerates have similar chemical composition to that of ettringite. The CNMs agglomerates are properly placed in terms of ettringite formations due to the high quantities of accumulated water. The ettringite crystals have a needle-shaped, brittle, and porous structure, which can act as a starting point for micro-cracks, and consequently reduce physical performance. In contrast, the incorporation of an optimum concentration of hybrid CNTs and GNPs can prevent nano- and micro-scale crack propagation via their bridging mechanism. Furthermore, these CNMs increase the required energy of crack expansion by the deviation of its path or direction (Figure 11).

### 3.3. Cement Hydration Evaluation

**Figure 12:**
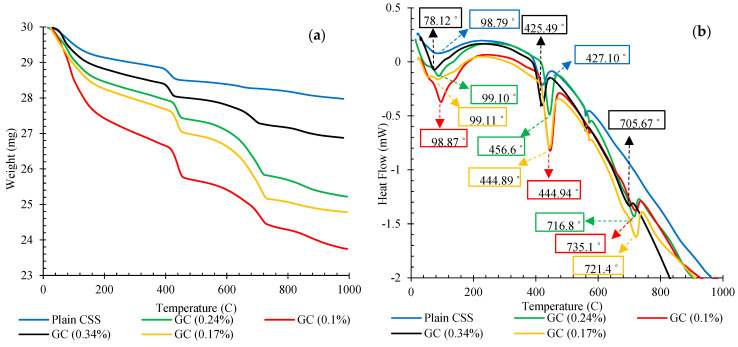
Thermal analysis of plain and reinforced CSS by different concentrations of CNMs: (**a**) TGA thermograms, (**b**) DSC thermograms.

Tricalcium silicate (Ca_3_SiO_5_, abbreviated C_3_S), dicalcium silicate (Ca_2_SiO_4_, C_2_S), tetra calcium aluminoferrite (Ca_4_Al_n_Fe_2-n_O_7_, C_4_AF), tricalcium aluminate (Ca_3_Al_2_O_6_, C_3_A), and small amounts of gypsum (CaSO_4_.2H_2_O) and clinker sulfate (Na^2-^SO_4_, Ka_2_SO_4_) are the main components of the chemical composition of cement grains in the anhydrous state [27]. C_3_A, C_3_S, C_4_AF, and C_2_S react with water following specific chemical reactions during the hydration process to produce monosulfonate (AFm), hydrate of calcium silicate (C-S-H) gel, ettringite (AFt), and calcium hydroxide (CH) [41,42]. During these reactions, the formation of CH, C-S-H, AFt, and AFm with various geometries and crystal shapes, as the most influential hydration products, plays a key role in determining the final properties of the cement and, eventually, through the evolution of the hydration process, they form a dense microstructure [43]. One of the major techniques used for the investigation of cement hydration products is thermogravimetric analysis (TGA). The results of TGA tests are illustrated in Figure 12a. In addition, the results of differential scanning calorimetry (DSC) have been presented in Figure 12b in order to better observe the temperature range associated with each weight decay. A range of weight losses for the powder of cementitious composite samples was observed in the TGA curves between 105 and 1000 °C, as found in previous studies [44,45].

From the TGA thermogram, the first decrease was up to 105 °C, which can be attributed to the elimination of sample-free water and moisture of CSS. Due to the dehydration of chemically bound water existing in hydration materials such as ettringite, C-A-S-H, carbo-aluminates, and C-S-H, the second decline in the TGA curves occurred between 105 and 400 °C. The next significant decline was detected between 400 and 550 °C, which can be linked to the CH dehydroxylation. The fourth and final decay was due to calcium carbonate, and clinker carbonation was found between 600 and 800 °C [46,47].

Results showed that the incorporation of an optimum concentration of CNMs into the CSS increased the hydration products and specifically raised the amount of the CH and C-S-H gel [48,49]. The maximum quantity of cement hydration products was achieved by the addition of 0.1% hybrid CNT/GNP, and an excessive increase in the CNM concentration caused the reduction in hydration.

The positive effects of CNMs on the cement hydration process have been reported in previous studies [50,51]. CNMs can increase and accelerate the formation of numerous cement hydration products owing to their nucleation agent effects and their surficial functional groups, thereby resulting in strong bonding performance between CNMs and the cementitious matrix [27]. Comparing the results of this study with previous studies that used similar CNMs shows that, in samples made by compaction method, the higher amount of hydration products was obtained in a lower dosage of this nanomaterial. This can be directly related to the reduction in water content in this fabrication method [27].

It should be noted that the excessive increase in CNM concentration absorbs the water needed for the cement hydration process, which reduces the hydration products and, in some cases, stops the hydration process of the cement. As mentioned in this study, PF-127 was used in order to disperse the CNMs. The molecular structure of the PF-127 is composed of a central hydrophobic chain of polyoxypropylene (PPO) and two hydrophilic chains of polyoxyethylene (PEO) placed on two sides. The PPO chains are absorbed on the surface of the CNMs during specific reactions and PEO chains also trap water molecules on the surface and between layers of CNMs and their agglomerates [28].

**Figure 13:**
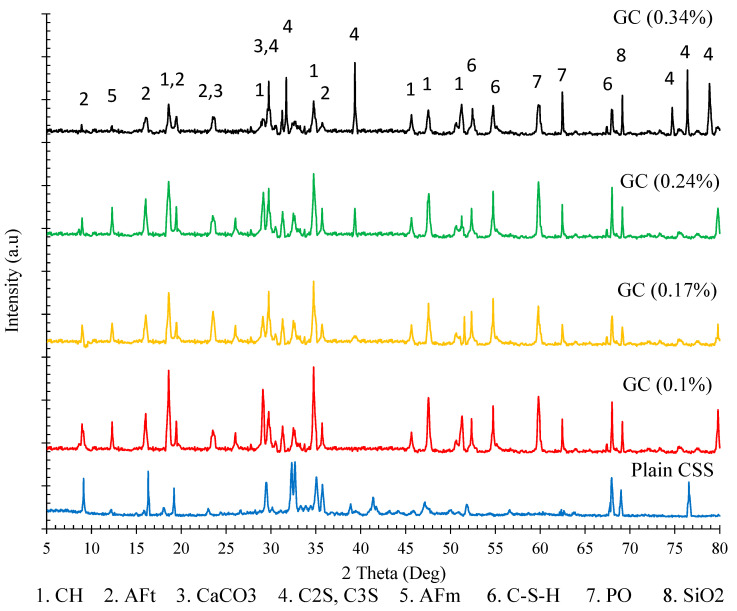
X-ray diffraction analysis (XRD) patterns of hardened plain and reinforced CSS by different CNM concentrations.

The XRD analysis result of hardened plain CSS and CSS reinforced by different CNM concentrations, presented in Figure 13, also showed a similar trend. As can be observed, the amount of the hydration product was increased by the incorporation of a certain concentration of CNM (0.1%), while an excessive increase in CNMs caused a reduction in CH and C-S-H, in addition to increasing unhydrated cement components, such as C2S and C3S.

### 3.4. Mechanical Characterizations

**Figure 14:**
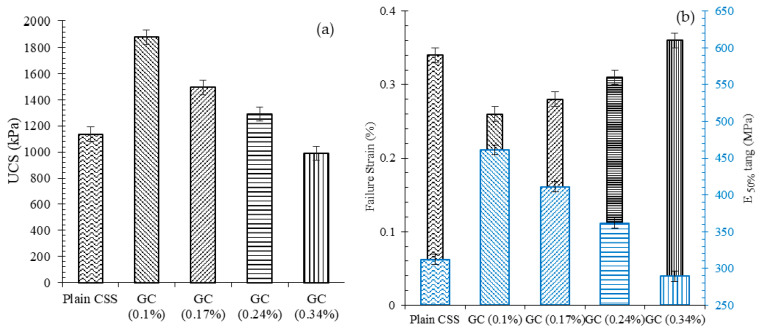
(**a**) Unconfined compressive strengths of different CSSs, (**b**) the amount of strain at the rupture point and E_(50%)_ after 28 days of hydration.

The results of axial unconfined compressive strengths, in addition to the axial strain at failure (maximum stress) and the tangent modulus at 50% of the peak stress (E _(50%)_), for plain and reinforced CSS, by different CNM concentrations, are illustrated in Figure 14a,b.

As can be seen, the incorporation of 0.1%, 0.17%, and 0.24% CNMs into the CSS caused an increase in UCS of about 65%, 31%, and 14%, respectively, compared to the plain CSS, while an excessive increase in the CNM concentration beyond 0.24% to 0.34% led to a reduction in UCS by around 13%.

Furthermore, the results showed that reinforcing CSS by specific CNM concentrations caused reductions in the ductility of specimens, such that the addition of 0.1%, 0.17%, and 0.24% CNM increased the E _(50%)_ of plain CSS by about 48%, 32%, and 16%, respectively. The specimen GC (0.34%), which was composed of a high CNM concentration, did not show a noticeable change in the amount of rupture modulus.

Concerning the failure strain, a similar trend was observed. However, the incorporation of 0.1%, 0.17%, and 0.24% CNM into the CSS reduced the maximum strain by around 25%, 19%, and 9%. An excessive increase in the CNM concentration up to 0.34% caused an increase in maximum strain of about 6%. Indeed, reinforcing cementitious composites by CNMs reduced their ductility and increased their stiffness.

Correia et al. [37] also reported an increase in UCS and E _(50%)_ by around 65% and 145%, respectively, for clayey sand, which compacted cement and MWCNT by 11% and 1%, respectively.

### 3.5. Durability Investigation

**Figure 15:**
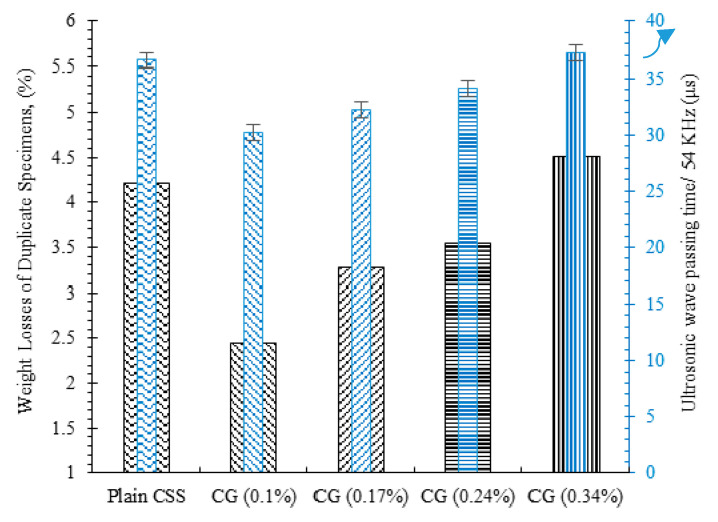
Weight loss and ultrasonic wave passing time after 12 climatic cycles.

The percentage of the specimens’ weight loss and their ultrasonic wave passing time after 12 climatic cycles, as the criteria of the specimens’ resistance and durability against the climatic cycle, is illustrated in Figure 15. As can be observed, the plain specimen showed a weight reduction of around 4.2%, whereas this amount for specimens composed of 0.1%, 0.17%, and 0.24% CNM was 2.4%, 3.2%, and 3.5%, respectively. These results show the positive effects of CNMs in terms of durability augmentation of cemented sand against harsh climatic cycles. However, increasing the concentration of the CNMs beyond 0.1% caused a reduction trend for the specimens’ resistance to the climatic cycle, in such a way that the incorporation of 0.34% CNM caused a greater weight loss than that of the plain specimen, equal to 4.51%. The dense and highly cohesive microstructure of the CSS caused by the intrusion of specific CNM concentrations increased the resistance of the sample to decay, contractions, and expansions caused by the climatic cycle. The results of the ultrasonic non-destructive tests also show a similar trend. The ultrasonic wave passing time along the longitudinal axis of the plain specimen was around 35.5 μs after 12 cycles, whereas the obtained passing times for the specimens containing 0.1%, 0.17%, 0.24%, and 0.34% CNM were 30.1, 32.2, 34, and 37.1 μs, respectively.

### 3.6. Electrical Behavior Investigation

#### 3.6.1. Electrical Conductivity

**Figure 16:**
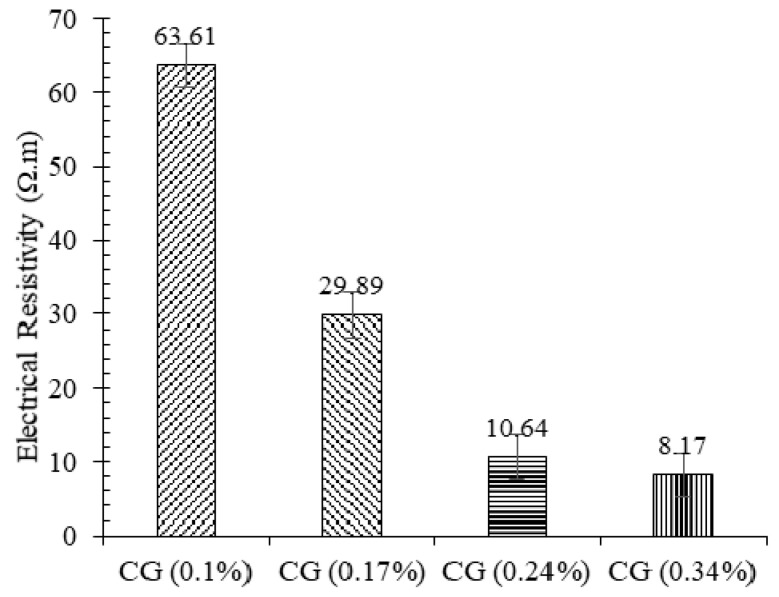
Electrical resistivity of hardened plain and reinforced CSS by different CNM concentrations.

The electrical resistivity of plain and reinforced CSS by different CNM concentrations is shown in Figure 16. As expected, the electrical conductivity of the CNM-reinforced specimen increased significantly due to the conductive path formation by percolation and electron tunneling mechanisms.

Increasing the carbon concentration continuously increased the conductivity of the composite in such a way that the electrical resistivity of specimens composed of 0.1%, 0.17%, 0.24%, and 0.34% CNM decreased by around 46%, 74%, 92%, and 95%, respectively, compared to the plain CSS. According to these results, the percolation threshold for this type of reinforced CSS could be around 0.24% due to the insignificant effect of CNM on reducing electrical resistivity in increments beyond 0.24%. By comparing the results with other studies that used similar types of sand, cement, dispersion method, and CNM, in order to investigate piezoresistivity and electrical conductivity of cementitious mortar (W/C = 0.5) [5,28], it can be observed that decreasing the cement concentration in CNM-reinforced cementitious composite led to the reduction in electrical resistivity. Comparing these results also showed that the use of a compaction fabrication method can reduce the electrical resistivity by up to 80%.

#### 3.6.2. Effects of Water Content on Electrical Conductivity

**Figure 17:**
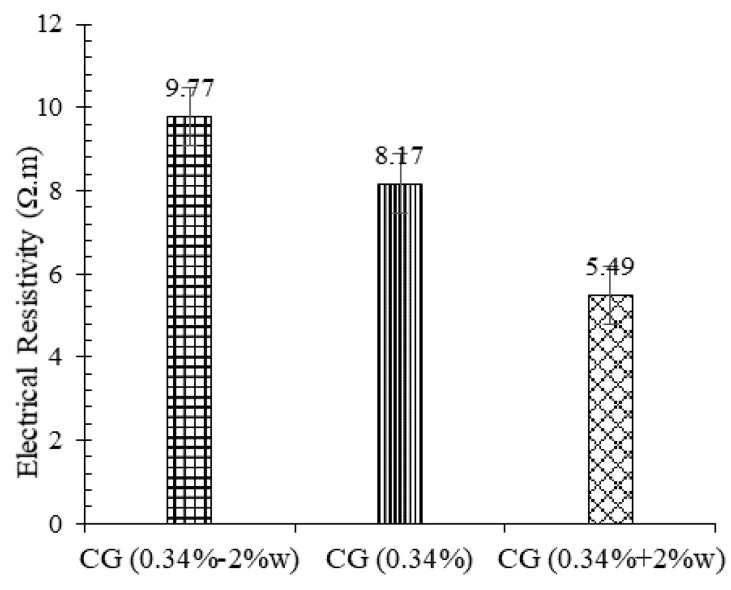
The electrical resistivity of the specimen CG (0.34%) at optimum, 2% more than optimum, and 2% less than optimum water content.

The electrical resistivity of the specimens with different water content and the same CNM concentration are shown in Figure 17. These specimens were composed of 0.34% CNM and fabricated with optimum, 2% more than optimum, and 2% less than optimum water content.

As shown in Figure 17, the electrical resistivity of the specimens containing 2% more water than the optimum concentration was reduced by around 33% compared to the specimens fabricated at the optimum water content, while, for the specimen composed of 2% less water than the ω_opt_, the electrical resistivity was increased by about 20%. Generally, increasing the water content assists CNM dispersion, which causes an increase in electrical conductivity [52,53,54]. In addition, increasing the water content increased the formation of ettringite (AFt) crystals, which are filled with water molecules and charged ions. These needle-shaped crystals also increase the electron tunneling effect and percolation mechanism, which reduce the electrical resistance.

#### 3.6.3. Effects of Climatic Cycles on Electrical Conductivity

**Figure 18:**
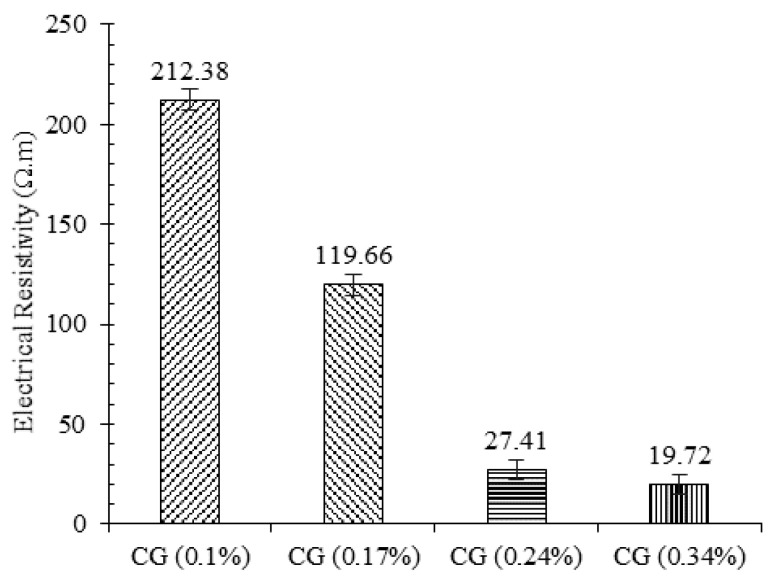
Electrical resistivity of the reinforced specimens with different concentrations of the CNM after climatic cycles.

The electrical resistivity of the reinforced specimens with different concentrations of CNM after climatic cycles is shown in Figure 18. By comparison with the results of the electrical resistivity of the specimens before climatic cycles (Section 3.6.1), it is clear that the electrical conductivity of the samples drastically decreased following the climatic cycles.

As can be seen, the electrical resistivity of the specimens composed of 0.1%, 0.17%, 0.24%, and 0.34% CNM increased by around 5, 3, 1.5, and 1.4 times, respectively, compared to the normal specimens. The destructive effects of wetting–drying combined with freeze–thaw cycles on the conductive system, including CNMs and free charged ions, are the major reason for the decrease in the electrical conductivity of the composite.

**Figure 19:**
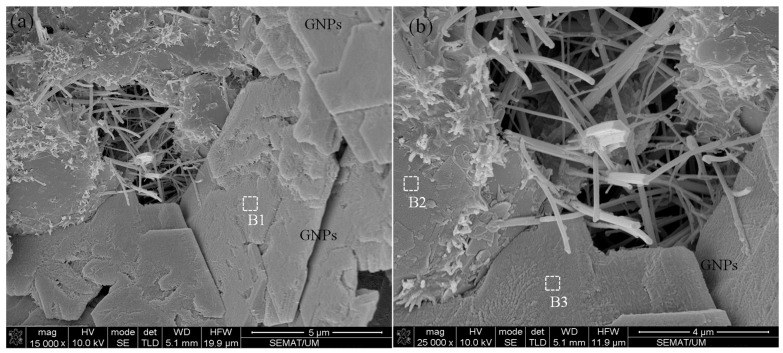
(**a**,**b**) CNM morphologies after climatic cycles (the areas with white markings are the areas selected for EDX analysis).

The morphology of CNMs after climatic cycles at different magnifications is illustrated in Figure 19. The SEM images clearly indicate the surficial structure of GNPs suffered from relatively severe degradation and damage. The surface texture of GNPs and their layers disintegrated due to the destructive effects of hybrid wetting–drying and freeze–thaw cycles, which caused a reduction in the electrical conductivity of the composite. In addition, EDX analysis was performed for CNMs, and their chemical compositions are listed in Table 4.

The results show that the quantity of carbon element in GNP chemical compounds was greatly reduced by climatic cycles compared to the normal specimens (Table 3, Position A4). However, increasing the oxygen concentration is evidence of GNPs’ excessive oxidation, which also caused GNPs to be surrounded by other elements and ions, such as Fe, Mg, Ca, Si, S, Al, and K, which can eliminate the free capacity of the π-electron in GNPs and CNTs. It should be noted that the π-electron plays as a crucial rule in the electrical conductivity of the CNMs [55,56].

**Figure 20:**
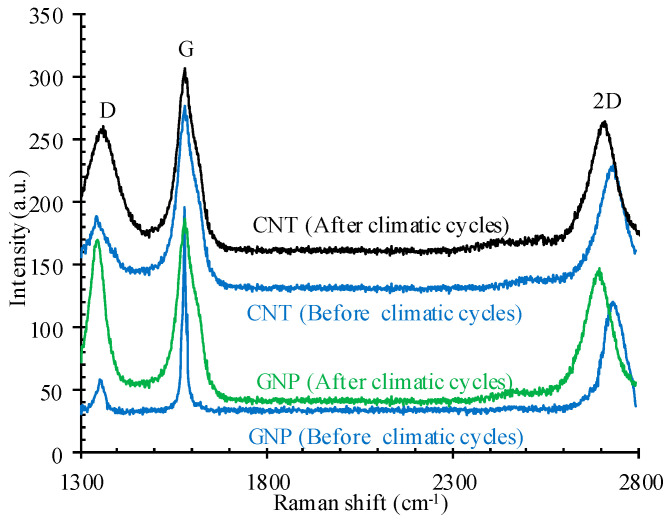
Raman spectra of the CNMs after climatic cycles.

Raman spectroscopy was also carried out on CNTs and GNPs in order to more accurately investigate the CNMs after climatic cycles; its analysis results are shown in Figure 20.

In the Raman spectra of CNTs and GNPs, the D band refers to the presence of defect concentrations and disturbances in C-C bonds, which are also referred to as non-sp^2^ (sp^3^) structural defects. Indeed, in the perfect hexagonal sp^2^-based carbon crystal structure, the D band is not as high, but, owing to the disturbance of the aromatic system, it is active for defective and small layers [31,57].

The G band in GNPs and CNTs defines a graphitic crystallinity state that can be ascribed to the C-C bonds’ tangential vibration (sp^2^ hybridization) and upshift to high wave numbers under the chaotic state [58]. Ultimately, the 2D band is an overtone of the defect-related D band and purity grade of the sample, which is specified with its corresponding intensity and broadening [59,60]. Hence, the variations in the characteristics of G, D, and 2D in Raman spectra represent the evolution of structural specification [31]. As can be seen, the intensity of the D and 2D band in both CNMs and GNPs after hybrid climatic cycles was significantly increased, which testifies to the presence of significant (sp^3^) structural defects and the reduction in the sp^2^ domain (La). This can greatly reduce the inherent electrical conductivity of CNTs and GNPs [31].

#### 3.6.4. Piezoresistivity Investigations

**Figure 21:**
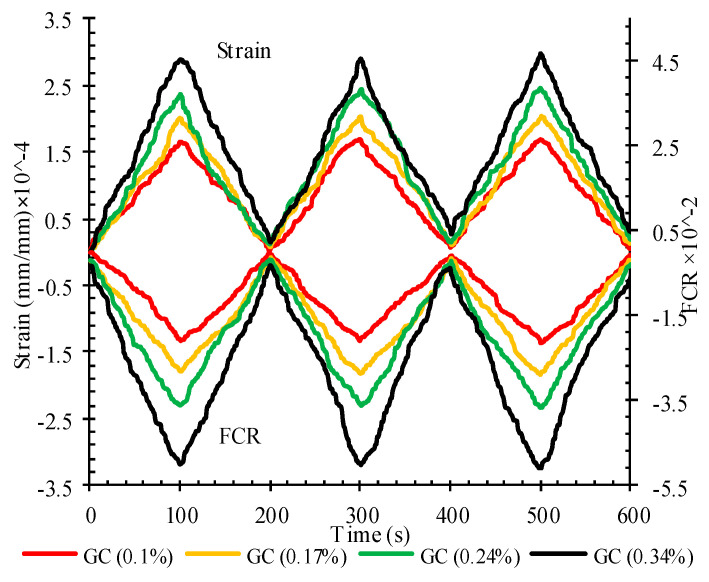
The fractional change in resistivity and axial strain under cyclic compression loading for reinforced CSS by different CNM concentrations.

The fractional change in resistivity against the axial strain under the cyclic compression loading for reinforced CSS by different CNM concentrations is indicated in Figure 21.

As can be seen, the negative values were achieved for FCR. In compression loading, the CNM particles and conductive paths became closer to each other, which consequently decreased the second resistance and caused negative values for FCR following Equation (4). The FCR amounts did not reach zero at the end of each loading cycle and a negligible amount of FCR remained after unloading, which is consistent with the strain remaining at the end of each loading cycle, and was increased by increasing load cycles. These remaining amounts were lower than those of previous studies [5] due to the compaction fabrication methods.

**Figure 22:**
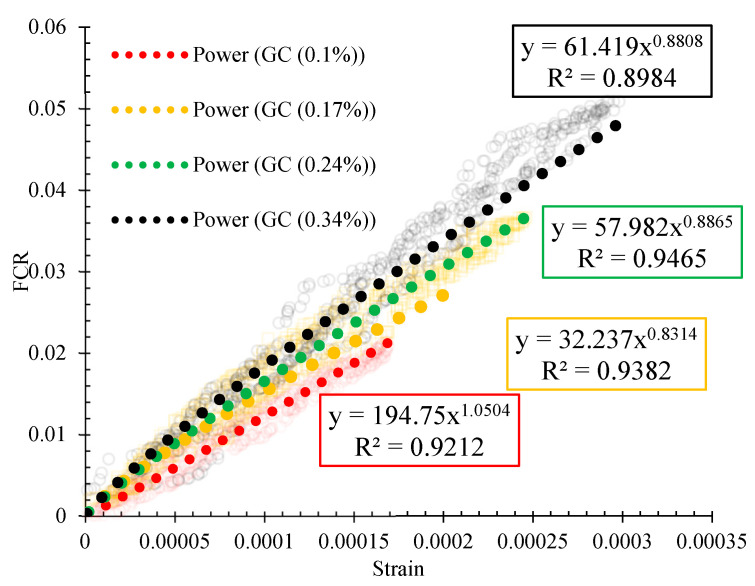
Variation of strain with the FCR for reinforced CSS by different CNM concentrations.

The variation of the strains with the fractional changes in resistivity for reinforced CSS by different CNM concentrations is also illustrated in Figure 22. A power function with a proper approximation was utilized to express the relationship between strain and FCR. Generally, the slopes of the diagrams consist of two parts. Although the slopes of the first part were increased by the increase in the CNM concentration, the specimens composed of lower CNM dosage showed higher slopes in the second part.

By increasing the CNM concentration beyond the percolation threshold, the sensitivity of the composites against the high amount of stress and/or strain was decreased due to the saturation of the composite structure from the conductive paths. By comparing these results with previous studies, it can be seen that the scatters of the data have been decreased [5].

#### 3.6.5. Effects of Water Content on Piezoresistivity Performance

**Figure 23:**
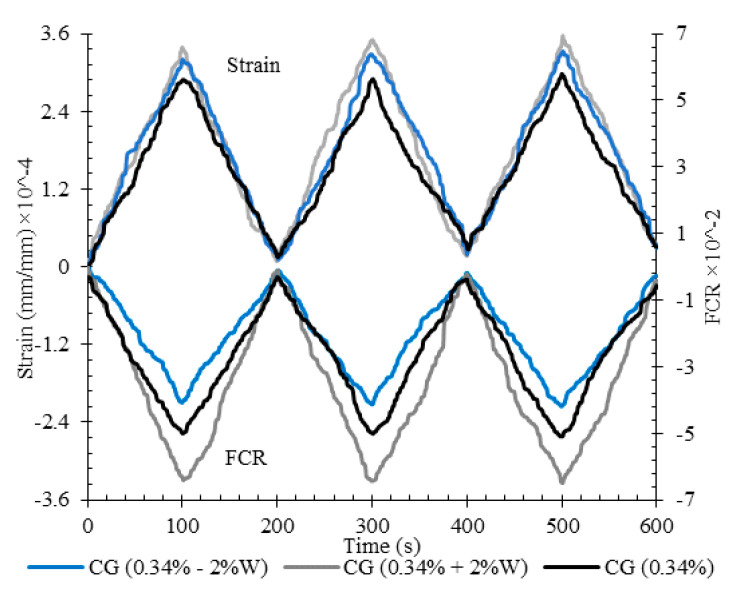
The fractional change in resistivity together with axial strain under cyclic compression loading for specimen CG (0.34%) at optimum, 2% more than optimum, and 2% less than optimum water content.

The fractional changes in electrical resistivity vs. the axial strain for specimens CG (0.34%) fabricated with ±2% water relative to the optimum water content, under the cyclic compression loading, have been shown in Figure 23.

**Figure 24:**
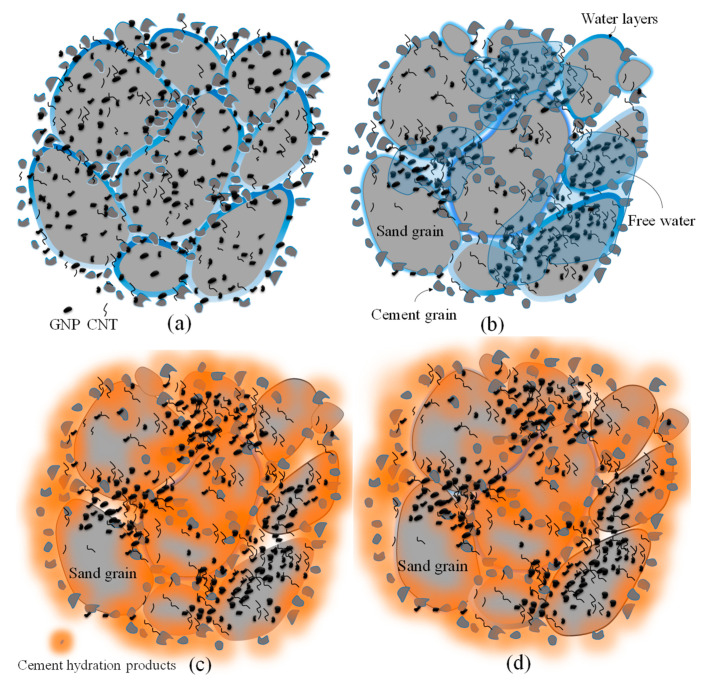
A schema of the compacted specimen composed of the CNMs: (**a**) fresh sample at optimum water content, (**b**) fresh sample at the higher or lower water content, (**c**) hardened specimen at the higher or lower water content, (**d**) hardened specimen at the higher or lower water content, subjected to the compression loading.

The results showed that fractional changes in electrical resistivity were increased by increasing the water content, while decreasing the water content caused a reduction in fractional changes in electrical resistivity. As mentioned, increasing the water content, in addition to the enhancement of the dispersion of nanoparticles and the improvement of conductive behavior by ionic conduction, also increases the amounts of strain due to higher amounts of porosities among the microstructure of the specimen (Figure 24). Hence, the conductive paths are more affected, which eventually leads to more fractional changes in the electrical resistivity.

Although, in specimens composed of lower water content, the grains are not well locked together and the amount of strain is also larger due to the high amount of porosity, the fractional changes in electrical resistance are lower due to the poor ionic conduction and dispersion of CNMs. As indicated in Figure 24, increasing the water concentration until the optimum increases the density of the samples, and a decrease in density is subsequently observed. The presence of the extra water content between the grain causes gaps between them and finally leads to porosities formation. By increasing effective stress or after drying, the free water will be eliminated and ultimately cause the strain to increase. In the case of lower water content, the lack of water required for lubrication prevents grains from locking properly in each other, which finally leads to the formation of porosity and increased strain when the specimen is subjected to the load. However, in low water content, the formation of the porous and needle-shaped crystals of ettringite is lower, which can lead to lower strain compared to the higher water content cases.

**Figure 25:**
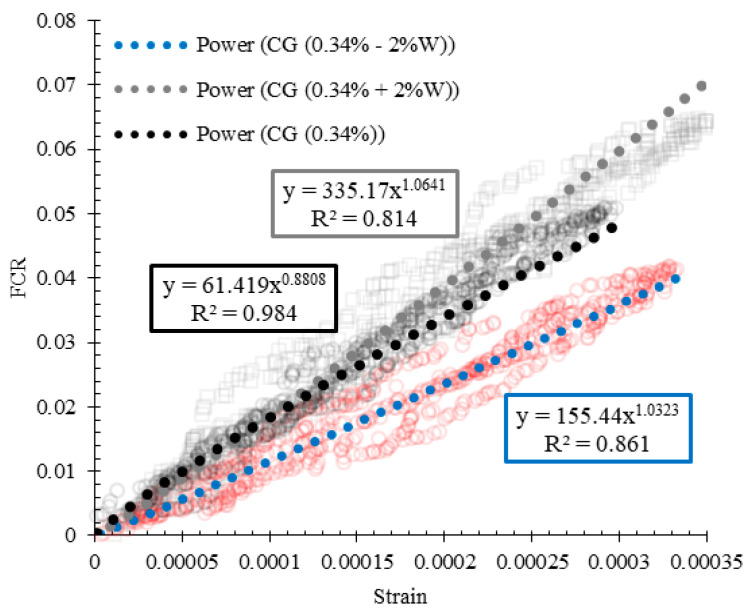
Variations of strain with the FCR for specimen CG (0.34%) at optimum, 2% more than optimum, and 2% less than optimum water content.

The variations of strain against the fractional changes in electrical resistivity for the specimen containing 0.34% CNMs at optimum, 2% more than optimum, and 2% less than optimum water content, have been illustrated in Figure 25.

As can be seen, the slope of the diagram for the specimen composed of higher water content is steeper than specimens fabricated at optimum and lower water content, which is evidence of its higher sensitivity. Additionally, the data scatter of the specimen CG (0.34%) is lower compared to the other specimens. Besides, the specimens that contained higher water content showed higher data scatter, which may be due to its porous microstructure.

#### 3.6.6. Effects of Climatic Cycles on Piezoresistivity Performance

**Figure 26:**
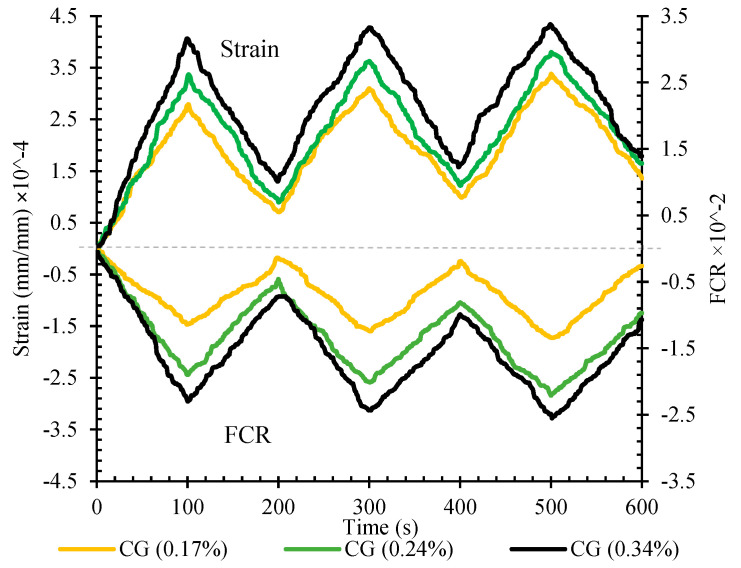
The fractional change in resistivity together with axial strain under cyclic compression loading for reinforced CSS by different CNM concentrations after climatic cycles.

The fractional change in resistivity vs. strain for nano intruded specimens after climatic cycles have been shown in Figure 26. As can be seen, the specimen CG (0.1%) did not show the piezoresistivity behavior after climatic cycles. In addition, the fractional changes in electrical resistivity of other specimens were decreased significantly. However, the amounts of their strain at the peak of loading and end of the loading showed significant growth. Despite such circumstances, increasing the CNM concentration also caused an increasing of the fractional changes in electrical resistivity, as well as peak and residual strains.

Generally, the destructive effects of the climatic cycles can be categorized into two main groups of adverse effects on composites’ microstructure, and inherent properties of CNMs. The effects of successive expansion due to water freezing along with the decay of hydration products due to frequent wetting–drying cycles, reduce the cohesiveness of the composite microstructure and cause its deterioration, as well as the formation of nano cracks.

**Figure 27:**
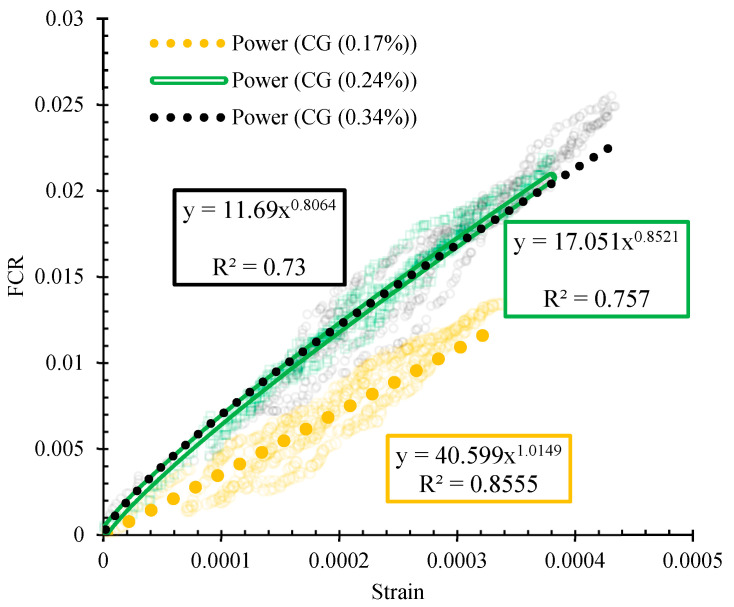
Variation of strain with the fractional changes in electrical resistivity (FCR) for nano-intruded specimens after climatic cycles.

This feeble matrix with defective microstructure does not have the required strength to maintain conductive paths and by increasing the load a sudden interruption occurred in conductive paths. Meanwhile, as mentioned in Section 3.6.3, the electrical conductivity of the CNMs has been extremely reduced due to the severe weather cycle. The relation between fractional changes in electrical resistivity of specimens and this axial strain after climatic cycles, under compression loading, has been illustrated in Figure 27.

As shown, increasing the CNM concentration resulted in a steeper slope. However, the slopes of the specimens composed of 0.24% and 0.34% CNM were almost equal. Furthermore, the scattering of the data was increased compared to the normal nano-intruded specimens (Figure 22).

#### 3.6.7. Gauge Factor

**Figure 28:**
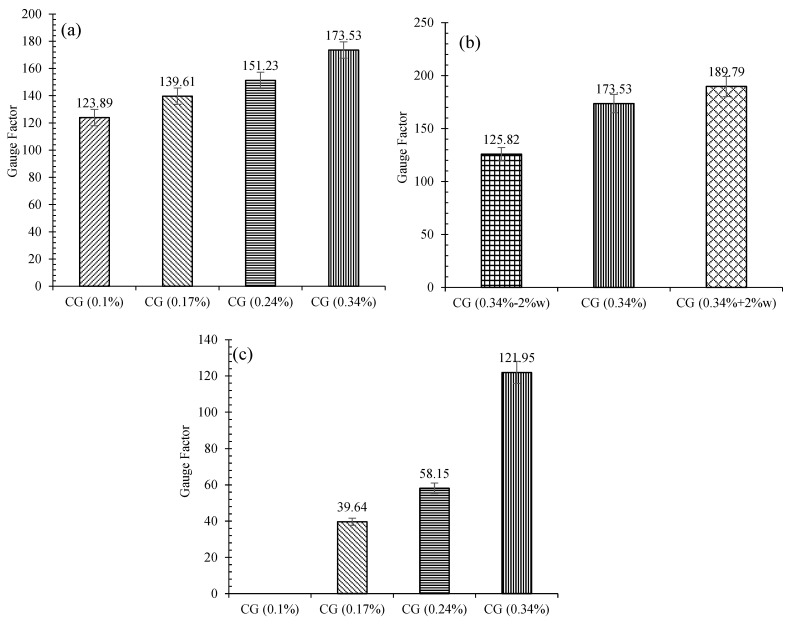
Variation of CNM reinforced specimens’ gauge factors under compression loading: (**a**) in normal condition, (**b**) with different water content, (**c**) after climatic cycles.

The variation of the gauge factors for reinforced CSS with different CNM concentrations and water content, in normal conditions and also after climatic cycles, is shown in Figure 28. The results indicate that, in normal conditions, increasing the CNM concentration raised the gauge factors, which shows the sensitivity improvement of the specimens against the strain. Generally, increasing the CNM concentration from 0.1% to 0.17%, 0.24%, and 0.34% led to the enhancement of the gauge factor by around 13%, 22%, and 40%, respectively.

Comparing these results with previous studies, which used hybrid CNT/GNP for reinforcing mortar [5], showed that the use of the compaction fabrication method can somewhat reduce the sensitivity and gauge factor.

As shown in Figure 28b, increasing the water content increased the gauge factor; increasing the water content by 2% more than optimum content increased the gauge factor of specimen CG (0.34%) by around 10%, whereas reducing the water by 2% compared to the optimal value reduced the gauge factor by 34%.

The investigation of the specimens’ gauge factors after climatic cycles (Figure 28c) clearly shows that the gauge factors decreased compared to those of the normal case due to the destructive effects of the climatic cycle. Indeed, the gauge factors of the specimens composed of 0.17%, 0.24%, and 0.34% CNM were reduced by 72%, 61%, and 30%, respectively, compared to the normal specimens.

## 4. Conclusions

This study was a systematic effort to develop a novel self-sensing cementitious geocomposite (cementitious stabilized-sand (CSS)), based on piezoresistivity behavior, with high physical and mechanical performance.

The series of tests carried out on specimens with different concentrations of hybrid CNTs and GNPs (1:1) dispersed into cementitious stabilized sand (with cement constituting 10% of the dry weight of the sand), compacted at the optimum water content, revealed that 0.1% was the optimum concentration of carbon nanomaterials to achieve the best mechanical behavior (strength and stiffness), the maximum electrical resistance and enhanced durability of the geocomposite against severe climatic cycles, including freeze-thaw and wetting–drying. These results were explained by the microstructure of the geocomposite (SEM, EDX, XRD, and TGA tests) and showed that the combination of 1D and 2D carbon nanomaterials (CNMs), in addition to filling the nano and micro porosities, can bridge and/or deviate the cracks, which can prevent their expansion. However, reinforcing CSS by CNMs generally increased the modulus and, consequently, reduced the ductility of the composite. Although reinforcing the CSS by 0.1% hybrid CNT/GNP increased the rate of the cement hydration due to their nucleation effects, excessive increases in the CNM concentration reduced hydration products due to the required water absorption of the hydration process. Furthermore, the Raman spectroscopy and piezoresistivity investigations indicated that the harsh climatic cycles negatively impacted the inherent electrical properties of the CNMs and, consequently, the sensitivity of the composite.

The outcomes of this study clearly showed the significant benefits of the compaction method, which is representative of transport and energy infrastructure works, in terms of mechanical and piezoresistivity performance enhancement, compared with mixing procedures typically used for cementitious composites.

In summary, we believe that this novel approach contributes to the new era of smart composite materials in intelligent structures.

## Figures and Tables

**Table 1:** Characteristics of graphene nanoplatelets (GNPs) and carbon nanotubes (CNTs).

GNP										
Surface Area (m^2^ g^−1^)	Density (g/cm^3^)	Carbon Content (%)	Tensile Modulus (Gpa)	PH Value (30 °C)	Tensile Strength (GPa)	Layers	Dimension		Form	Part Number
120–150	0.6	>99.5	1000	7–7.65	5	10 < n < 60	**Thickness**	**Diameter**	Gray Powder	TGN201
							4–20 nm	5–10 µm		
**MWCNT**										
**Surface Area (m^2^ g^−1^)**	**Density (g/cm^3^)**	**Color**	**Outside Diameter (nm)**	**Length (µm)**	**Ash (wt%)**		**Carbon Content (%)**	**Part Number**		
350	0.27	Black	<8	30–10	<1.5		>98	GCM327		

**Table 2:** Sand particle size distribution.

**Mesh Size (mm)**	0.08	0.16	0.5	1	1.6	2
**Cumulative retained (%)**	99 ± 1	87 ± 5	67 ± 5	33 ± 5	7 ± 5	0
**Specific gravity G_s_**	2.67		**Cu ^a^**	7.5	**Cc ^b^**	1.8

^a^ Uniformity coefficient, ^b^ curvature coefficient.

**Table 3:** Cement hydration crystal chemical composites.

Position	Elements (%)									
	C	O	Ca	Al	Si	S	Mg	K	Fe	P
Figure 10 (A1)	8.72	36.53	35.81	3.67	10.47	1.29	0.52	0.19	0.29	0.61
Figure 10 (A2)	11.29	36.59	33.23	4.57	12.01	1.11	0.27	0.37	0.15	0.41
Figure 10 (A3)	9.3	38.44	31.73	6.18	11.71	1.95	0.43	0.11	0.27	0.24
Figure 10 (A4)	81.31	4.27	7.91	-	5.72	-	-	-	-	0.79

**Table 4:** CNMs’ chemical composition.

Position	Elements (%)									
	C	O	Ca	Al	Si	S	Mg	K	Fe	P
Figure 19 (B1)	29.46	37.61	15.7	4.81	8.15	2.64	0.71	0.14	-	0.83
Figure 19 (B2)	34.51	35.18	14.64	3.19	7.12	3.91	0.29	0.18	0.27	0.71
Figure 19 (B3)	32.77	39.41	11.92	3.89	7.51	3.24	0.42	0.12	0.15	0.58

## Data Availability

Requests for all types of data used to support the findings of this study, after the publication of this article, will be considered by the corresponding author, subject to obtaining permission from the owners.
